# Influence of Whey Protein Micro-Gel Particles and Whey Protein Micro-Gel Particles-Xanthan Gum Complexes on the Stability of O/W Emulsions

**DOI:** 10.3390/polym13142301

**Published:** 2021-07-14

**Authors:** Man Zhang, Bin Liang, Hongjun He, Changjian Ji, Tingting Cui, Chanchan Sun

**Affiliations:** 1 College of Life Sciences, Yantai University, Yantai 264005, China; 20172905850@m.ldu.edu.cn (M.Z.); hemiles@ytu.edu.cn (H.H.); 2 College of Food Engineering, Ludong University, Yantai 264025, China; liangbin1989311@163.com; 3 Department of Physics and Electronic Engineering, Qilu Normal University, Jinan 250200, China; sdejcj@qlnu.edu.cn; 4 Key Laboratory of Food Nutrition and Safety, Tianjin University of Science & Technology, Ministry of Education, Tianjin 300457, China; tingtingcui@sdas.org

**Keywords:** whey protein concentration micro-gel particles, xanthan gum, stability, O/W emulsion

## Abstract

Appropriate pretreatment of proteins and addition of xanthan gum (XG) has the potential to improve the stability of oil-in-water (O/W) emulsions. However, the factors that regulate the enhancement and the mechanism are still not clear, which restricts the realization of improving the emulsion stability by directional design of its structure. Therefore, the effects of whey protein micro-gel particles (WPMPs) and WPMPs-XG complexes on the stability of O/W emulsion were investigated in this article to provide theoretical support. WPMPs with different structures were prepared by pretreatment (controlled high-speed shear treatment of heat-set WPC gels) at pH 3.5–8.5. The impact of initial WPC structure and XG addition on Turbiscan Indexes, mean droplet size and the peroxide values of O/W emulsions was investigated. The results indicate that WPMPs and XG can respectively inhibit droplet coalescence and gravitational separation to improve the physical stability of WPC-stabilized O/W emulsions. The pretreatment significantly enhanced the oxidative stability of WPC-stabilized O/W emulsions. The addition of XG did not necessarily enhance the oxidative stability of O/W emulsions. Whether the oxidative stability of the O/W emulsion with XG is increased or decreased depends on the interface structure of the protein-XG complex. This study has significant implications for the development of novel structures containing lipid phases that are susceptible to oxidation.

## 1. Introduction

Oil-in-water (O/W) emulsions such as salad dressing, soups, sausages, and mayonnaise represent important systems in the food industry. Their high stability, including storage stability and oxidative stability, is the basis of other functional properties. Therefore, a variety of emulsifiers including macromolecular proteins, polysaccharides and low molecule weight surfactants are introduced to stabilize the emulsions and inhibit lipid oxidation.

Whey protein (WP) has been widely used as emulsifier in various emulsion products such as mayonnaise and ice cream, which attributed to its high nutritional quality and excellent functional properties [1]. Many studies have confirmed that an interfacial WP layer may inhibit lipid oxidation due to its ability to form a physical barrier between the continuous phase and dispersed phase, chelate pro-oxidant transition metals and scavenge free radicals [2]. However, WP-based emulsions are very sensitive to destabilization, especially when the temperature and pH are changed. Moreover, lipid and protein co-oxidation lead to instability of the emulsions, decreased sensory properties and nutritional values [3]. Thus, steps must be taken to improve the stability of the WP-stabilized emulsions. The functional properties of proteins can be enhanced using many techniques such as heating, ultrasound, high-pressure treatment, and cross-linking with the usage of different enzymes [4]. It can be seen that proper pretreatment can enhance the stability of protein-stabilized emulsions. Our previous studies found that the controlled high-speed shear treatment of heat-set WP gel could significantly enhance the emulsifying properties and emulsifying stability of WP [5]. However, the influence of the controlled high-speed shear treatment of heat-set WP gel on the stability of WP-stabilized emulsion is still unclear.

Xanthan gum (XG) is one of the most important functional biopolymers added to O/W emulsions, and used as stabilizer due to its thickening and gelling properties [6]. However, due to its poor interfacial activity, XG is rarely used alone, but rather in combination with other emulsifiers in emulsions. Recent studies have shown that both the physical stability and oxidative stability of O/W emulsions stabilized by proteins can be enhanced by adding XG [7,8]. They concluded that this could be attributed to the increased thickness of the interfacial layer, the changing rheological properties of the emulsions and the ability to chelate metal ions of XG. However, the results of Griffin and Khouryieh [9] showed that at every NaCl concentration, the secondary lipid oxidation products (TBARS) concentration of WP-XG emulsion at pH 7 was significantly higher than that of other groups. Thus, the effect of XG on stability of protein-stabilized emulsions is not always enhancement, depending on some factors. However, the effect factors and mechanism are not clear, which restricts the realization of enhanced stability by the fine design of O/W emulsion structure.

The interactions between polysaccharides and proteins depend on the distribution of functional groups (e.g., charged and hydrophobic, etc.) and environmental conditions (e.g., pH and biopolymer ratios, etc.) [10]. Under the influence of the environmental conditions and the distribution of various functional groups, protein/polysaccharide mixtures in an aqueous dispersion are often accompanied by either segregative/associative phase separation through thermodynamic incompatibility or compatibility [11]. Therefore, the functional properties and structure of protein-polysaccharide complex emulsifiers can be regulated by the modification of protein structure.

In this article, WPC micro-gel particles (WPMPs) with different molecular structures were prepared by the controlled high-speed shear treatment of heat-set WPC gels at pH 3.5–8.5. The impact of XG addition on the short-term stability, mean lipid droplet size and the peroxide values (PVs) of WPC and WPMPs-stabilized O/W emulsions was investigated to determine whether WPMPs and WPMP-XG complexes-stabilized emulsions would provide better physical and oxidative stability than WPC and WPC-XG complexes-stabilized emulsions. The differences of the emulsifiers (proteins and protein-XG complexes) structure were characterized by a Rheolaser Lab micro-rheometer based on multi-speckle diffusing wave spectroscopy (DWS). The influence of whey protein micro-gel particles and whey protein micro-gel particles-xanthan gum complexes on the stability of O/W emulsion was evaluated by using Turbiscan Lab^®^ Expert stability analysis. By exploring the correlation between the differences of the initial structure of proteins and protein-XG complexes and emulsion stability, the article will provide theoretical support for the directional design of food emulsion structure to improve its stability.

## 2. Materials and Methods

### 2.1. Materials

Whey protein concentrate (WPC) powder was supplied by Fonterra Commercial Trading Co., Ltd. (Shanghai, China). The composition of WPC as specified by the manufacturer was 80.3% protein (wet basis), 6.9% carbohydrate, 5.1% moisture, 3.8% fat, and 3.9% ash. XG (Ziboxan^®^TW) was of analytical grade and obtained from Deosen Co., Ltd. (Shanghai, China). Soybean oil was obtained from COFCO Co. Ltd. (Beijing, China). Methanol, chloroform, NaOH, HCl, ferrous chloride, and potassium thiocyanate (analytical grade) were purchased from Sinopharm Co. Ltd. (Beijing, China).

### 2.2. Whey Protein Micro-Gel Particles Preparation

WPC powder (12 g) was dispersed in 100 mL distilled water by stirring for 2 h at 25 °C to ensure complete hydration of the proteins. And then, the pH of the WPC dispersions were separately adjusted to pH 3.5, pH 4.5, pH 5.5, pH 6.5, pH 7.5, and pH 8.5 using 0.1 mol/L HCl or 0.1 mol/L NaOH solution. The dispersions were thermally-crosslinked by heating at 85 °C for 16 min in a water bath. And then, the samples were cooled with cold water (0 °C) for 10 min and held at 4 °C for 10 h. After homogenizing with an Ultra-Turrax T25 high-speed homogenizer (IKA, Staufen, Germany) at 10,000 r/min for 4 min, the pH of the dispersions was all adjusted to pH 6.5 using the above NaOH and HCl solution. In order to eliminate the influence of Na^+^ concentration on the results, 1 mol/L NaCl solution was used to make the concentration of Na^+^ equal in each test sample. Suspensions of WPC micro-gel particles (WPMP) were obtained and named WPMP(3.5), WPMP(4.5), WPMP(5.5), WPMP(6.5), WPMP(7.5) and WPMP(8.5).

### 2.3. Particle Size Distribution Measurements of WPMPs

Immediately after preparation, particle size distributions of WPC and WPMP suspensions were respectively measured using dynamic light scattering-based particle size analyzers (BT-9300S and BT-2001, Dandong Bettersize Instruments Ltd., Dandong, China) at 25 °C. The laser analyzer is based on dynamic light scattering (DLS). The Stokes-Einstein equation was used to calculate the particle size and distribution.

### 2.4. WPMP-XG Complexes Preparation

Powdered XG (1 g) was dispersed in 30 mL MilliQ water and then stirred at 80 °C for 30 min. Subsequently, the WPMP dispersions and XG solution (7:3, *w*/*w*) were mixed by stirring (AHM-P125A hand-held mixer, Appliance Company of America (Zhuhai) CO., LTD, Zhuhai, China) at 200 r/min for 5 min. The complexes of XG and WPMP(3.5), WPMP(4.5), WPMP(5.5), WPMP(6.5), WPMP(7.5), WPMP(8.5) were named WPMP(3.5)-XG, WPMP(4.5)-XG, WPMP(5.5)-XG, WPMP(6.5)-XG, WPMP(7.5)-XG and WPMP(8.5)-XG, respectively. The ultimate ratio of protein: XG is 7.5:1 (*w*/*w*) in the protein-XG complexes.

### 2.5. Micro-Rheology Measurements of WPMP Dispersions and WPMP-XG Complexes

A commercial Rheolaser Master analyser (Formulaction Inc., l’Union, France), used to measure the micro-rheological properties of WPMP dispersions and WPMP-XG complexes, is based on diffuse wave spectroscopy (DWS). Immediately after preparation, 20 mL of liquid samples were placed into flat-bottomed cylindrical glass tubes (height of 140 mm, diameter of 16 mm). The temperature of all the samples was set to 25 °C. And then, the first measurement of the backscattered light was made. Micro-rheological properties, including the elasticity index (EI), macroscopic viscosity index (MVI), solid-liquid balance (SLB) and fluidity index (FI), were obtained from the mean square displacement curves [12].

### 2.6. Emulsion Preparation

Emulsions containing 20% (*w*/*w*) soybean oil were prepared with soybean oil and WPMP dispersions and WPMP-XG complexes using a high-shear homogenizer IKA Ultra-Turrax T25 (IKA) at 10,000 r/min for 5 min at 25 °C.

### 2.7. Short-Term Stability Measurement of Emulsions

Immediately after preparation, 20 mL of liquid samples were placed into flat-bottomed cylindrical glass tubes (height of 140 mm, diameter of 16 mm). And then, the stability of all the emulsions was evaluated using Turbiscan Lab^®^ Expert stability analysis (Formulaction) and multiple light scattering principle [13]. The Turbiscan Stability Indexes (TSI) as a function of short-term (24 h) were calculated according to backscattered light intensity values [14]:

(1) $$\mathrm{TSI}=\sqrt{\frac{\sum_{i-1}^{n}(x_{i}-x_{BS)}}{n-1}}$$

where, $x_{i}$ is the backscattered light intensity values of every scan; $x_{BS}$ is the mean of backscattered light intensity values; n is the number of scans.

### 2.8. Mean Droplet Size of Emulsions

For backscattered light intensity, it is also affected by droplet size and volume concentration of oil. Thus, the droplet size as a function of short-term (24 h) can be calculated according to the following formula:

(2) $$\mathrm{BS}=\sqrt{\frac{3\emptyset \left(1-g\right)Q_{s}}{2d}}$$

where, BS is the backscattered light intensity values; ∅ is the volume concentration of oil; g and $Q_{S}$ are the optical parameters based on Mie theory; d is the mean droplet size, nm.

### 2.9. Determination of the Peroxide Values (PVs) of the Emulsions

The emulsions stabilized by WPMP and WPMP-XG complexes were stored at 30 °C for 17 d. Every 24 h, 2 g samples of each emulsion were placed in a flask and dissolved with 6 mL of a mixture of trichloromethane/methanol (7:3, v:v) [15]. After vortexing three times, the mixture was centrifuged at 10,000× *g* for 5 min. 0.2 mL of the extract (lower layer) was removed to measure the PVs.

The PVs was determined according to the method outlined in GB/T 5009.37-2003 [16] with a slight modification. The extract was diluted with 4.8 mL or 9.8 mL of trichloromethane/methanol (7:3, v:v). The mixtures were then reacted with 15 μL of ferrous chloride mixed solution (0.03 mol/L ferrous chloride and 0.5 mol/L HCl) and 15 μL of 0.3 mol/L potassium thiocyanate solution. After full mixing with vortex, the samples were incubated for 5 min at 25 °C in the dark. The absorbances were measured at 500 nm using a TU-1810 ultraviolet spectrophotometer (Persee General Instrument Co., Ltd., Beijing, China). The PVs (expressed as milligram equivalent of hydroperoxide per kilogram of oil) was quantified according to Equation (2):

(3) $$\mathrm{PV}\;(\mathrm{meq}/\mathrm{kg})=\frac{C-C_{0}}{0.6\times m\times D\times 55.94\times 2}$$

where C and $C_{0}$ are the iron contents (μg) of the emulsions and blank, respectively, which were calculated based on the standard curve; *m* is the weight of the emulsions, g; and D is a dilution factor, 75 or 150.

### 2.10. Statistical Analysis

All measurements were performed a minimum of three times and the resulting datas were expressed as mean ± standard deviation (SD). Statistical analyses were performed by the Tukey post hoc test with one-way ANOVA, with *p* < 0.05 considered significant.

## 3. Results and Discussion

### 3.1. Particle Size Distribution of WPMPs

**Figure 1:**
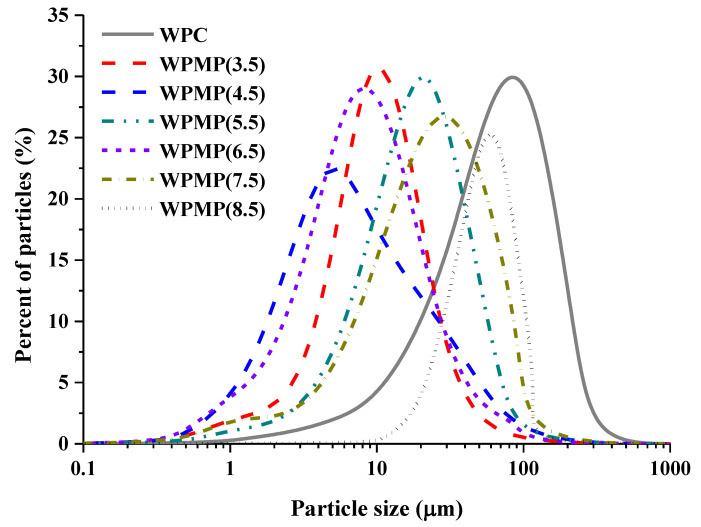
Particle size distributions of WPC and WPMPs.

As shown in Figure 1, all the particle sizes of WPC and WPMPs presented a normal distribution (unimodal distribution). Al the samples also had a narrow size distribution. Thus, the protein size can be characterized by mean particle size (*D*_50_) as shown in Table 1. WPC is a group of globular proteins with *D*_50_ of about 45.86 μm. *D*_50_ of WPMPs which were formed by controlled thermal denaturation of WPC at pH 3.5, pH 4.5, pH 5.5, pH 6.5, pH 7.5 and pH 8.5 were 7.54 μm, 4.91 μm, 13.96 μm, 6.19 μm, 17.74 μm, and 46.68 μm. In the pH range of 3.5–5.5, with the increase of pH, particle sizes of WPMPs decreased first and then increased gradually. As the pH continued to rise from 5.5 to 8.5, the *D*_50_ of WPMPs gradually increased as the pH increased. The results are consistent with the research of Chen et al. [17]. They believed that the difference in particle size of protein gel particles is due to the redistribution of inner structure of protein molecules during heat treatment at different pH and cooling process. According to Liu et al. [18], the network structure and texture in a heat-induced whey protein gel largely depends on the balance between attractive and repulsive forces among denatured protein molecules during aggregation which effected by the pH values of the dispersions. Thus, the larger particle size of WPMP(5.5) than WPMP(4.5) and WPMP(6.5) may be due to the higher hardness of heat-set WPC gel formed at pH 5.5 than those formed at pH 4.5 and pH 6.5 [19], which is harder to be destroyed during the controlled high-speed shear treatment.

The SSA of WPMP(4.5), WPMP(5.5), WPMP(6.5), and WPMP(7.5) steeply increased to 0.40 m^2^/g, 0.58 m^2^/g, 0.24 m^2^/g, 0.52 m^2^/g, 0.25 m^2^/g, which respectively were five times, seven times, three times, six times and three times the SSA of WPC. Higher SSA may lead to differences in stability of the O/W emulsions stabilized by the proteins. *D*_50_, *D*[4,3] and SSA of WPMP(8.5) shows no significant difference with these of WPC. However, *D*[2,1] and *D*[3,2] significantly increased to 31.05 μm and 39.84 μm, which were bigger than these of WPC (4.19 μm and 23.83 μm). It indicates that the shape of WPMP(8.5) is no longer a spheroid. The results are consistent with our previous observations [20]. In addition to particle size, the shape of protein micro-gel particles is also an important parameter for its adsorption on the oil-water interface, thus affecting the stability of the emulsions [21].

### 3.2. Micro-Rheology Measurements of WPMP Dispersions and WPMP-XG Complexes

#### 3.2.1. EI and MVI of Samples

The viscoelasticity of the emulsifiers (WPMP dispersions and WPMP-XG complexes) is represented by EI and MVI values. Ceniti et al. [22] indicated that the EI corresponds to the inverse of the distance to be covered by particles before interacting with the network, and the MVI corresponds to the inverse of the speed of the particles over long distances. EI values measure elastic features of the emulsifiers, and are linked to end-use properties such as mesh size and hardness of the network. MVI values quantify the microscopic viscosity of the emulsifiers at rest, and can be proportional to the macroscopic viscosity.

**Figure 2:**
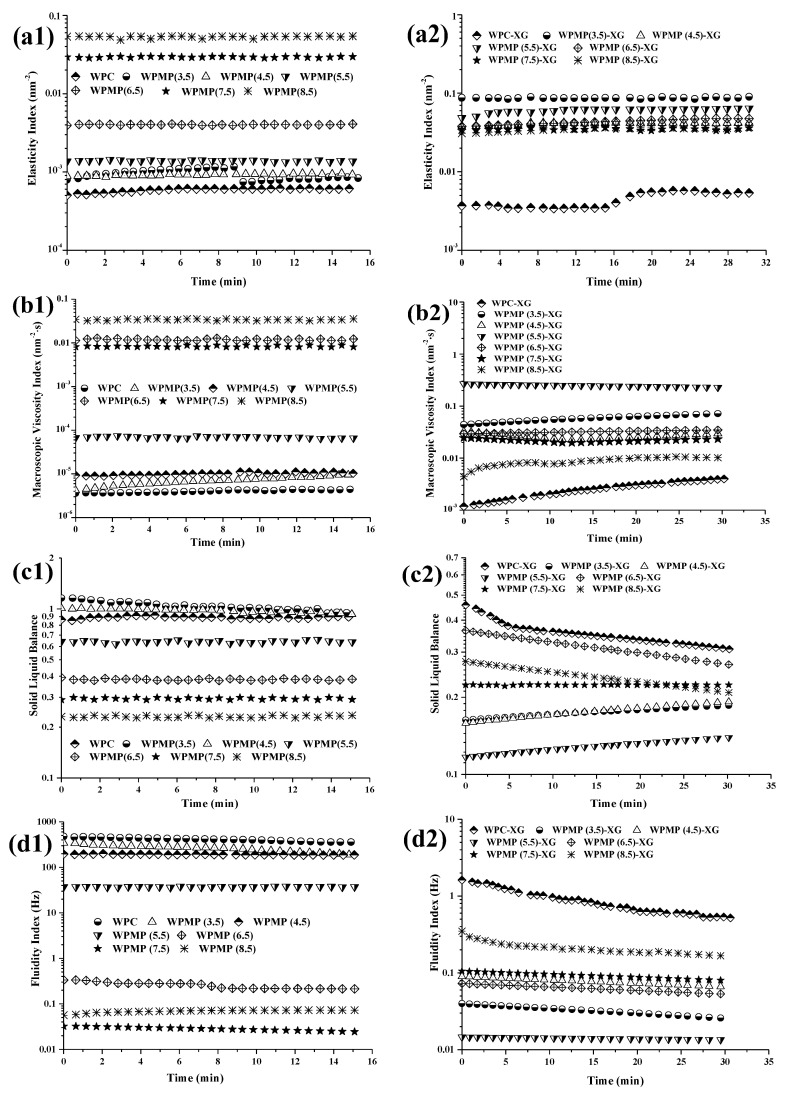
Micro-rheological properties of the proteins (1) and protein-XG complexes (2). (**a1**,**a2**): Elasticity index; (**b1**,**b2**): Macroscopic viscosity index; (**c1**,**c2**): Solid liquid balance; (**d1**,**d2**): Fluidity index.

As can be seen from Figure 2(a1,b1), the EI and MVI values of WPMP dispersions were significantly higher than those of WPC dispersions. The results indicate that the structure and rheological properties of WPC changed during the preparation of WPMPs at different pH levels. In the pH range of 4.5–8.5, with the increase of pH, EI and MVI values of WPMPs increased gradually. Protein solutions with high protein concentrations show two behavioral stages during heating, namely molecular unfolding and aggregation [23]. In the first stage, that is, the protein molecular unfolding stage, the protein molecules overcome the intra-molecular force and unfold under the action of solvent (water molecules) and heat treatment. At the same time, the embedded free sulfhydryl and hydrophobic groups are exposed. In the second stage, the unfolded protein molecules are cross-linked with each other to form soluble/insoluble aggregates through hydrogen bonding, hydrophobic interactions and S-S crosslinking. Different pH values of dispersions significantly affect the charges of protein molecules. Thus, in the first stage, the extent of protein unfolding, and the distribution of amino acid side chains were significantly different. Thus, the network strength and particle size of the protein aggregates formed in the second stage vary significantly with the pH values of the dispersions. Subsequently, the controlled high-speed shearing treatment destroys the heat-set protein gel and forms protein micro-gel particles [5]. When the pH approaches the isoelectric point (~pH 4.5), the protein molecules have less charge and the intermolecular electrostatic repulsion is weaker than other pH values. Thus, it is easy to form random micro-aggregates through non-covalent protein-protein interactions [24] which can be easily destroyed into small protein micro-gel particles. The weak interaction between protein miacro-gel particles led that the EI and MVI values of WPMP(3.5), WPMP(4.5) and WPMP(5.5) significantly decreased as the pH approached the pI. As the pH value increases from pH 5.5 to pH 8.5, the viscoelasticity of protein miacro-gel particles significantly increased. The formation of compact protein aggregates at pH 6.5–8.5 could be a major contributor to the high EI and MVI values seen in WPMP(6.5), WPMP(7.5) and WPMP(8.5).

Figure 2(a2,b2) show that the EI and MVI values significantly increased after the addition of XG. WPC-XG showed the lowest EI and MVI values. In addition, WPMP(5.5)-XG, and WPMP(3.5)-XG exhibited higher EI and MVI values than WPMP(4.5)-XG, WPMP(7.5)-XG, WPMP(pH 6.5)-XG, and WPMP(8.5)-XG. Thus, it can be concluded that XG formed stronger networks with WPMP(5.5) and WPMP(pH 3.5). The pH values of WPMP dispersions were all pH 6.5, which is higher than the isoelectric point of the whey proteins (α-lactalbumin, pH 4.3; β-lactoglobulin, pH 4.7; and bovine serum albumin, pH 5.2). At this time, whey proteins and XG are negatively charged, so the intermolecular repulsion is dominant. According to Bryant & Mcclements [25], phase separation which is driven by thermodynamic incompatibility occurred between the proteins and polysaccharides in the protein-XG complexes in the neutral pH range. This is consistent with the research of Laneuville et al. [26] who found that low WP: XG ratios (5:1–10:1) produced principally small incompatibility complexes at the pH value higher than pI.

It is noteworthy that the viscoelasticity of WPMPs-XG complexes is significantly different. According to our previous research results [27], the unfolding of the native conformation of WPC exposed the hydrophobic amino acids and caused the concomitant burial of some hydrophilic amino acid side chains during the combined treatment of heat treatment and controlled high-speed shear treatment. Changes in the distributions of hydrophobic/hydrophilic amino acid side chains decreased the surface charge density of WPMPs. In WPMPS-XG complexes, the different surface charge density of WPMPs affected the degree of intermolecular repulsion between the WPMPs and XG, which eventually leads to the viscoelasticity difference of the complexes.

#### 3.2.2. SLB and FI of Samples

The SLB is directly proportional to the viscoelastic properties of the product and is indicative of the evolution of the ratio between the solid-like and liquid-like behaviour of the product as a function of time. The FI is obtained from the inverse of the characteristic decorrelation time which corresponds to the time needed to reach a relevant speckle pattern decorrelation. Thus, the SLB and FI can be used to compare the rheological behaviours of several samples [5]. SLB = 0 means that the sample is purely elastic/solid-like, 0 < SLB < 0.5 means that the solid behaviour is dominant (gel behaviour), SLB = 0.5 means that the liquid and solid behaviours are equal, and 0.5 < SLB < 1 means that the liquid behaviour is dominant [28]. A low FI (~10^−2^ Hz) means that the product is mainly solid-like, and a high FI (~10 Hz) means that product is mainly fluid-like.

As seen in Figure 2(c1,d1), the SLB and FI values of WPC, WPMP(3.5), WPMP(4.5) and WPMP(5.5) were greater than 0.5 and 10, respectively, indicating that the liquid behaviour dominates in each of these samples. WPC was heated and controlled by high-speed shearing to be used as building blocks to form various types of colloidal particles. The applied treatment reduces the proteins to very small spheroidal particles with a diameter of 4.91–13.96 µm. under acidic conditions (pH 3.5–5.5). The relative sliding between WPMP particles is the main reason for their good fluidity. The SLB and FI values of WPMP(6.5), WPMP(7.5) and WPMP(8.5) were less than 0.5 and 1, respectively, indicating that the solid behaviour is dominant. The resulting gels formed by heat treatment at pH above pH 6.5 are more translucent, clastic and rubbery, which were hard to break down or can restore structure quickly [29]. Therefore, the fluidity of the WPMPs was poor.

Figure 2(c2,d2) show that the SLB and FI values of all the protein-XG complexes were less than 0.5 and 10, respectively, indicating that the proteins and XG formed complexes are dominated by gel behaviour. According to our previous studies, XG formed network structures in the complexes, and the WPMPs were evenly dispersed among them [30]. The network blocked the relative sliding of WPMP particles, resulting in the decreasing SLB and increasing FI. The difference of SLB and FI depends on the structural uniformity of the protein-XG incompatibility complexes and the accumulation of WPMPs. The difference of surface charge density of WPMPs caused by the distributions of hydrophobic/hydrophilic amino acid side chains during the combined treatment of heat treatment and controlled high-speed shear treatment is the most fundamental reason.

**Figure 3:**
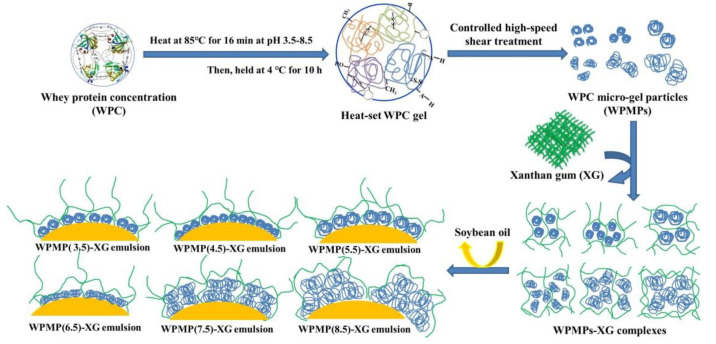
Hypothetic schematic representation of WPMPs, WPMP-XG complexes and interfacial structure of emulsions.

Based upon the results, the authors made a hypothetic schematic representation of the interaction and distribution of WPMPs and XG which were shown in Figure 3. In the protein-XG complexes, WPMP particles were aggregated and distributed among the stable three-dimensional network formed by XG molecules. The degree of WPMP particles aggregation from high to low was WPMP(8.5), WPMP(7.5), WPMPMP(6.5), WPMPMP(4.5), WPMPMP(3.5), and WPMPMP(5.5).

### 3.3. Analysis of Stability and Mean Droplet Size

**Figure 4:**
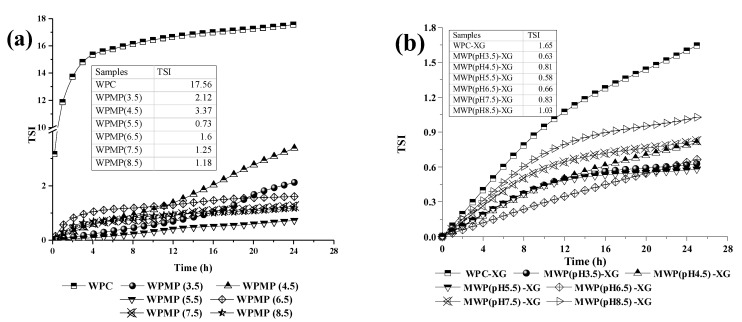
Turbiscan stability index (TSI) of the emulsions stabilized by the proteins (**a**) and protein-XG complexes (**b**).

**Figure 5:**
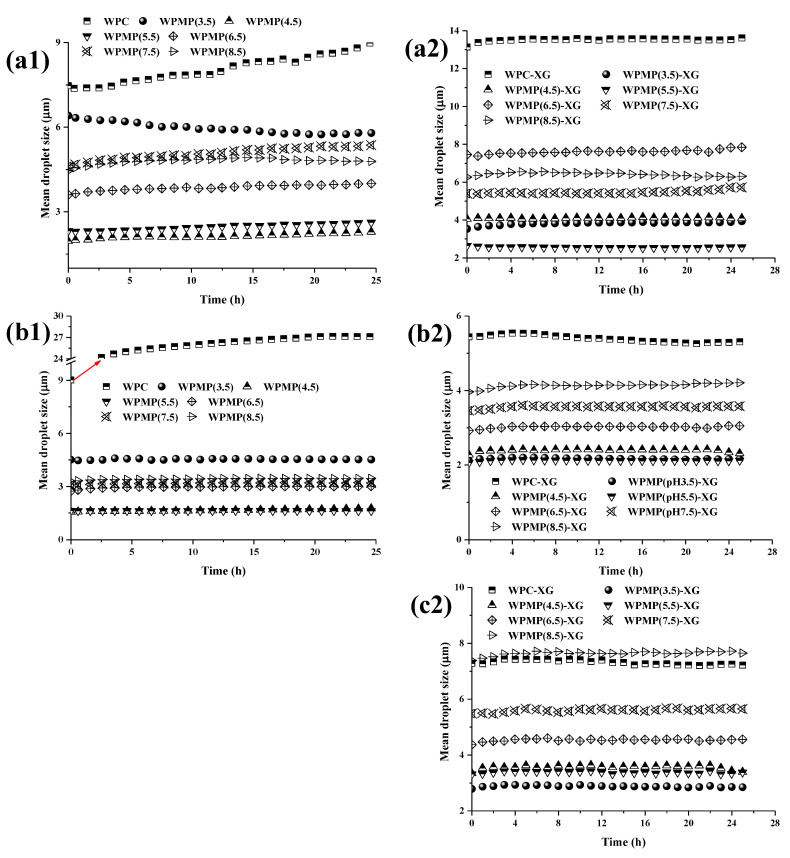
Mean droplet size of the emulsions stabilized by the proteins (1) and protein-XG complexes (2) during storage. (**a1**,**a2**) Mean droplet sizes on the top of the emulsions; (**b1**,**b2**) Mean droplet sizes in the middle of emulsions; (**c2**) Mean droplet sizes on the bottom of the emulsions. No corresponding values for mean droplet size measurements on the bottom of the protein-stabilized emulsions.

Figure 4 shows the TSI values of the emulsions stabilized by WPMP dispersions and WPMP-XG complexes during 24 h of storage at 25 °C. Smaller TSI values indicate higher stability. The emulsion stabilized by WPC was the worst among all the studied emulsion systems, which was evidenced by the remarkable increase in its TSI (up to 17.56). It can be seen from Figure 5(a1,b1) that with the prolongation of storage time in 24 h, the mean droplet size increased significantly at the top (from 7.5 μm to 9.0 μm) and in the middle parts (from 9.0 μm to 26.2 μm) of the WPC emulsion. This indicates coalescence behavior of oil droplets in the emulsion which is one of the reasons for a significant increase in TSI values. According to Evans, Ratcliffe, & Williams [31], the result was partly due to that the concentration of dissolved WPs surrounding the smaller droplets was higher than that of the larger droplets, resulting in a concentration gradient. Over time, the gradient made the dissolved molecules move from the smaller droplets to the larger droplets, resulting in the overall increase of droplet size. The TSI values of the WPMPs-stabilized emulsions were significantly smaller than that of the WPC-stabilized emulsion, indicating that the stability of the emulsion is enhanced. After 24 h of storage, the TSI values from high to low were WPMP(4.5) emulsion, WPMP(3.5) emulsion, WPMP(6.5) emulsion, WPMP(7.5) emulsion, WPMP(8.5) emulsion, and WPMP(5.5) emulsion (Figure 4a). Except for WPMP(4.5) emulsion and WPMP(3.5) emulsion, the TSI curves of other WPMPs-stabilized emulsions showed a slow rising trend and reached the highest value at 20 h of storage time. According to Figure 5(a1,b1), the mean droplet size at the top and in the middle of all WPMPs-stabilized emulsions did not change, suggesting that flocculation between droplets increased the TSI during the 24 h storage period. The absence of corresponding mean droplet size values at the bottom of all the protein-stabilized emulsions was due to the occurrence of gravitational separation. The results indicate that WPMPs improved the physical stabilize of WPC-stabilized O/W emulsions with high stability against coalescence.

The TSI values of protein-XG complexes-stabilized emulsions were significantly lower than that of protein-stabilized emulsions, indicating that the stability of the emulsions was enhanced by the addition of XG (Figure 4b). According to Figure 4a,b, the TSI values of the WPMP(3.5)-XG emulsion, WPMP(4.5)-XG emulsion, WPMP(5.5)-XG emulsion, WPMP(6.5)-XG emulsion and WPMP(7.5)-XG emulsion were significantly lower (0.50–0.85) than that of the protein stabilized emulsions (0.75–17.56), indicating the addition of XG enhanced on the emulsion stability. Under neutral conditions (pH 6.5) in emulsions, the charge incompatibility between WPMPs and XG played a central role in the stability during storage [32]. Therefore, the charge density on the surface of the WPMPs directly affects the charge incompatibility. Natural whey proteins are small globular proteins with defined molecular conformations. In addition, the hydrophilic groups are widely distributed on the surface, while the hydrophobic groups are buried in the interior of the protein. Thus, the strong electrostatic repulsion between the negatively charged WPMPs and XG in the WPC-XG complexes-stabilized emulsion leads to the rapid increase of TSI curves (Figure 4b). In addition, after the combined treatment of heat treatment and controlled high-speed shear treatment at different pH, the hydrophobic interaction of the WPMPs would enhance to varying degrees, while the electrostatic interaction with the XG would be weakened. Thus, the stability of WPMPs-XG complexes-stabilized emulsions would be significantly higher than that of WPC-XG complexes-stabilized emulsions.

Figure 5(a2–c2) show that the mean droplet size of protein-XG complexes-stabilized emulsions did not change significantly during short-term storage. This indicates that oil droplet flocculation is the main reason for the slow rise of TSI values of protein-XG complexes-stabilized emulsions. From the Stokes formula, it can be found that the settling velocity of oil droplets is proportional to the square of their diameter [33]. Therefore, the constant droplet size is the key factor to prevent coalescence of the oil droplets and delay phase separation. According to the micro-rheological properties, stability, and mean droplet size, it can be speculated that XG can prevent the instability of emulsions by shielding the active charged groups of whey proteins and reducing the collision rate among molecules by increasing the viscosity of aqueous phase [34,35]. Therefore, the results showed that XG addition can inhibit droplet coalescence and gravitational separation to improve the physical stability of WPC-stabilized O/W emulsions.

### 3.4. Measuring the PVs of the Emulsions

**Figure 6:**
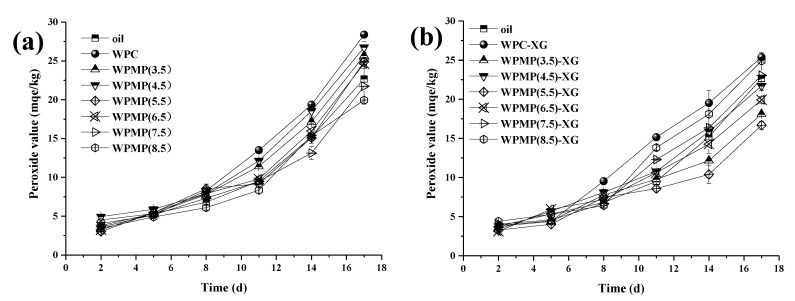
Peroxide values (PV) of the emulsions stabilized by the proteins (**a**) and protein-XG complexes (**b**) as a function of storage time.

Lipid oxidation is a process which is influenced by many factors, such as the lipid droplet size, emulsifier type and concentration [36]. The oxidative stability of the emulsions entails measurement of the levels of primary oxidation product generated during 17 days of storage. Hydroperoxides are the primary oxidation product and can be quantified via the PVs. Figure 6a shows the PVs of soybean oil and protein (WPC and WPMPs)-stabilized emulsions throughout a 17-day period. As the storage period progressed, the PVs of all emulsions significantly increased, indicating that all emulsions were oxidized. The relationship between the PVs (y) and storage time (x) was successfully fitted by the power-law model, y = a(1 + x)^b^ (Table 2). The power law indexes b of WPC emulsion, WPMP(3.5) emulsion, WPMP(4.5) emulsion, WPMP(5.5) emulsion and WPMP(6.5) emulsion are significantly greater than that of soybean oil (Table 2), indicating not only a greater rate but also a higher extent of oxidation. However, the b values of WPMP(7.5) emulsion and WPMP(8.5) emulsion were significantly lower than those of soybean oil, indicating good oxidation stability. The difference can be attributed to the dispersion of soybean oil in O/W emulsion into droplets in aqueous phase containing oxidants and oxygen [37]. Thus, the increased surface contact area caused high oxidative stress and promoted lipid oxidation in the O/W emulsions. Emulsions stabilized by WPMPs had significantly lower PVs than the WPC-stabilized emulsion (Figure 6a). The difference can be attributed to the aggregation and coalescence of lipid droplets in WPC-stabilized emulsion, which brought the lipid phases closer and facilitated the transfer of lipophilic pro-oxidants among the droplets. Notably, the PVs and b values of the WPMP(7.5) emulsion and WPMP(8.5) emulsion were significantly lower than those of the soybean oil after 17 days of storage, indicating that lipid oxidation in O/W emulsions can be inhibited by WPMP(7.5) and WPMP(8.5). The lipid droplet size is an important factor affecting the oxidative stability of O/W emulsions. With the increase of lipid droplet size, the PVs of emulsion significantly decrease and the oxidative stability of emulsions increases. However, PV results of WPMPs-stabilized emulsions were not exactly in the same order as that of the mean droplet size, indicating that the initial structure of the proteins was a more important factor affecting the oxidative stability of the O/W emulsions.

Figure 6b shows that the PVs of protein-XG complexes-stabilized emulsions decreased sequentially, WPC-XG emulsion, WPMP(8.5)-XG emulsion, WPMP(7.5)-XG emulsion, WPMP(4.5)-XG emulsion, WPMP(6.5)-XG emulsion, WPMP(3.5)-XG emulsion, and WPMP(5.5)-XG emulsion. The power-law indexes, b, were significantly smaller for WPC-XG emulsion, WPMP(3.5)-XG emulsion, WPMP(4.5)-XG emulsion, WPMP(5.5)-XG emulsion, WPMP(6.5)-XG emulsion than for the corresponding protein-stabilized emulsions (Table 2). And b values decreased in the order of WPMP(8.5)-XG emulsion, WPMP(7.5)-XG emulsion, WPC-XG emulsion, WPMP(4.5)-XG emulsion, WPMP(6.5)-XG emulsion, WPMP(5.5)-XG emulsion, and WPMP(3.5)-XG emulsion (Table 2). Compared with Figure 6a,b, it can be found that the PVs of emulsions stabilized by protein-XG complexes except WPMP(7.5)-XG and WPMP(8.5)-XG were significantly lower than those of protein-stabilized emulsions. This may be due to that XG forms a consecutive layer outside the protein interface, which further prevents the water-soluble oxidant and oxygen in the water phase from contacting with the lipid. The results are consistent with other previous studies [38,39,40] that the addition of XG can inhibit lipid oxidation in O/W emulsions. They found that it was attributed to that XG can chelate transition metals such as iron in the aqueous phase at neutral pH. In addition, they can increase the interfacial film thickness and provide a more robust barrier between dispersed phase and continuous phase.

However, PV values of WPMP(8.5)-XG emulsion were significantly greater than those of WPMP(8.5) emulsion (Figure 6a,b). This may be due to that WPMP(8.5) can form a dense protein adsorption layer on the surface of the lipid droplets, thus blocking the contact between water-soluble oxidant and oxygen in the water phase with the lipid phase. However, in WPMP(8.5)-XG emulsion, the protein adsorption layer was blocked by XG molecules into several discontinuous layers, providing contact sites for oxidant and oxygen (Figure 7). These results indicate that the effect of XG on the oxidative stability of O/W emulsion is also controlled by the interfacial structure of protein-XG. This finding indicates that the high oxidative stability of emulsion can be achieved by directional design of the interfacial structure of the protein-polysaccharide complexes. The PV results illustrate the potential applications of MWP(pH 7.5), MWP(pH 8.5), MWP(pH 3.5)-XG and MWP(pH 5.5)-XG as emulsifiers for the production of O/W emulsions containing lipid phases that are susceptible to oxidation.

## 4. Conclusions

Micro-gel pariticles with mean particle sizes (*D*_50_) of 7.54 μm, 4.91 μm, 13.96 μm, 6.19 μm, 17.74 μm, and 46.68 μm can be formed by controlled high-speed shear homogenization of heat-set WPC gel at pH 3.5–8.5. The micro-rheological properties indicated that low WP: XG ratios (7.5:1) principally produced low incompatibility complexes at pH values higher than pI. TSI curves and mean droplet size curves indicated that the short-term storage stability of WPC-stabilized emulsion was worse than that of other emulsions due to the occurred coalescence, flocculation, and gravitational separation. The increase of TSI values of WPMPs-stabilized emulsions was caused by flocculation, and gravitational separation. The reason for the rise of TSI curves of protein-XG stabilized emulsions is flocculation. Thus, WPMPs and XG can respectively inhibit droplet coalescence and gravitational separation to improve the physical stability of the emulsions.

PV results of WPMPs-stabilized emulsions were not exactly in the same order as that of the mean droplet size, indicating that the initial structure of the proteins was a more important factor affecting the oxidative stability of the protein-stabilized emulsions. Pretreatment (controlled high-speed shear homogenization of heat-set WPC gel) significantly enhanced the oxidative stability of O/W emulsions. The addition of XG does not necessarily enhance the oxidative stability of O/W emulsions. Therefore, whether the oxidative stability of O/W emulsions of XG addition is enhanced or weakened depends on the interfacial structure of the protein-XG complexes. Such WPMPs and WPMP-XG complexes exhibited an excellent capacity to prepare gel-like emulsions, and would be useful as functional ingredients to prepare emulsion-based products in the biotechnological, pharmaceutical, and food industries.

## Figures and Tables

**Table 1:** Particle size distribution of WPC and WPMPs.

Samples	*D*_50_ (μm)	*D*[2,1]	*D*[3,2]	*D*[4,3]	SSA * (m^2^/g)
WPC	45.86 ± 1.22 ^a^	4.19 ± 0.02 ^a^	23.83 ± 0.25 ^a^	50.90 ± 2.18 ^a^	0.08 ± 0.01 ^a^
WPMP(3.5)	7.54 ± 0.11 ^b^	1.80 ± 0.05 ^b,c^	4.83 ± 0.06 ^b^	8.39 ± 0.3 1^b^	0.40 ± 0.01 ^b^
WPMP(4.5)	4.91 ± 0.21 ^c^	1.52 ± 0.12 ^b,d^	3.36 ± 0.03 ^c^	8.32 ± 0.76 ^b^	0.58 ± 0.01 ^c^
WPMP(5.5)	13.96 ± 0.10 ^d^	2.12 ± 0.05 ^c^	7.91 ± 0.11 ^d^	15.91 ± 0.16 ^c^	0.24 ± 0.01 ^d^
WPMP(6.5)	6.19 ± 0.42 ^e^	1.42 ± 0.08 ^d^	3.71 ± 0.15 ^c^	8.78 ± 0.38 ^b^	0.52 ± 0.02 ^e^
WPMP(7.5)	17.74 ± 0.13 ^f^	1.51 ± 0.01 ^b,d^	7.75 ± 0.06 ^d^	21.99 ± 0.22 ^e^	0.25 ± 0.01 ^d^
WPMP(8.5)	46.68 ± 0.56 ^a^	31.05 ± 0.31 ^e^	39.84 ± 0.43 ^e^	48.91 ± 0.50 ^a^	0.05 ± 0.01 ^f^

The values that do not bear the same letter in the same column are significantly different (*p* < 0.05). *D*[4,3], *D*[3,2] and *D*[2,1] represent volume mean diameter, Sauter mean diameter and linear mean diameter, respectively, * Specific surface area.

**Table 2:** Relevant parameters of oxidative kinetic model.

Samples	y = a(1 + x)^b^		
	a	b	R^2^
oil	0.51	1.58	0.93
WPC	0.24	1.64	0.97
WPMP(3.5)	0.25	1.58	0.94
WPMP(4.5)	0.29	1.59	0.94
WPMP(5.5)	0.18	1.67	0.92
WPMP(6.5)	0.20	1.65	0.94
WPMP(7.5)	0.28	1.48	0.91
WPMP(8.5)	0.23	1.53	0.93
WPC-XG	0.48	1.37	0.98
WPMP(3.5)-XG	0.60	1.15	0.93
WPMP(4.5)-XG	0.46	1.32	0.97
WPMP(5.5)-XG	0.52	1.17	0.91
WPMP(6.5)-XG	0.51	1.25	0.96
WPMP(7.5)-XG	0.40	1.50	0.96
WPMP(8.5)-XG	0.41	1.51	0.95

## Data Availability

All the experimental data presented herein are available upon request from the corresponding author.
